# Identification and Analysis of Human Microbe-Disease Associations by Matrix Decomposition and Label Propagation

**DOI:** 10.3389/fmicb.2019.00291

**Published:** 2019-02-26

**Authors:** Jia Qu, Yan Zhao, Jun Yin

**Affiliations:** School of Information and Control Engineering, China University of Mining and Technology, Xuzhou, China

**Keywords:** microbe, disease, association prediction, matrix decomposition, label propagation

## Abstract

Studies have shown that microbes exist widely in the human body and are closely related to human complex diseases. Predicting potential associations between microbes and diseases is conducive to understanding the mechanisms of complex diseases and can also facilitate the diagnosis and prevention of human diseases. In this paper, we put forward the Matrix Decomposition and Label Propagation for Human Microbe-Disease Association prediction (MDLPHMDA) on the basis of the dataset of known microbe-disease associations collected from the database of HMDAD and the Gaussian interaction profile kernel similarity for diseases and microbes, disease symptom similarity. Moreover, the performance of our model was evaluated by means of leave-one-out cross validation and five-fold cross validation, and the corresponding AUCs of 0.9034 and 0.8954 ± 0.0030 were gained, respectively. In case studies, 10, 9, 9, and 8 out of the top 10 predicted microbes for asthma, colorectal carcinoma, liver cirrhosis, and type 1 diabetes were confirmed by literatures, respectively. Overall, evaluation results showed that MDLPHMDA has good performance in potential microbe-diseasepositive free parameter, which associations prediction.

## Introduction

Microbes are microscopic organisms that may exist in single-celled form or in a colony of cells (Madigan and Michaelt, 2015). They live in almost all the habitats from the poles to the deep sea and also make up the microbiota in all multicellular organisms (Delong and Pace, 2001). There are trillions of microbes in the human body. Lots of them are beneficial for human health, while others may cause infectious diseases (Thiele et al., 2013). Human microbiota can form an endosymbiotic relationship with their host, providing services and useful goods to humans. For example, the gut flora can contribute to gut immunity as well as digest complex carbohydrates and synthesize vitamins (O'hara and Shanahan, 2006). It is now accepted that most of the microbes are not intrinsically harmful. However, the pathogenic microorganisms and the imbalance of resident microbes are closely related to human disease.

Microorganisms are closely related to both infectious diseases and non-infectious diseases. Infectious diseases are global problems. They have induced several feared plagues in human history and new infections are still emerging today (Morse, 1995). Microorganisms are the causative pathogens for many infectious diseases. The involved organisms include pathogenic bacteria such as *Mycobacterium tuberculosis* and *Bacillus anthracis*, which can cause tuberculosis and anthrax, respectively (Hawn et al., 2014; Hendricks et al., 2014); protozoan parasites such as *Plasmodium* and *Toxoplasma gondii*, which can cause malaria and toxoplasmosis (Torgerson and Mastroiacovo, 2013; Iburg, 2015); and also fungi such as *Candida albicans* and *Histoplasma capsulatum* which can cause candidiasis or histoplasmosis (Stenn, 1960; Pappas et al., 2016). Meanwhile, most new infections appear to be caused by already discovered pathogenic microorganisms. These pathogens obtain selective advantage by changing conditions to infect new host populations or cause a new disease (Morse, 1991). On the other hand, microbiota can interact with human at multiple levels. Due to these complex microbiota-host relationships, dysbiosis can be the cause of the pathology (Forum on Microbial et al., 2014). Various factors including antibiotics, radiations, stress or nutritional changes can alter the compositions of human microbiota. This disruption of homeostasis can induce many maladies (Tamboli et al., 2004). For example, it is founded that the interactions between host immunity and gut microbiota can directly result in inflammatory bowel disease (IBD). IBD is a long-term aggravating inflammation of the intestine (Schirbel and Fiocchi, 2010). Both commensal microbiota and individual genetic susceptibility play key roles in the occurrence and development of this disease (Ferreira et al., 2014). Compared to healthy control, the composition of gut microbiota in IBD patients is distinct with decreased *Firmicutes* (Walker et al., 2011). The complex interplay between microbiota and human is also closely related to metabolic disease such as obesity (Ley et al., 2006). In a study about overweight and obese children, scientists found that the lower numbers of fecal *Staphylococcus aureus* was further linked with normal-weight development (Kalliomaki et al., 2008). Besides intestinal tract, microbial communities in respiratory tract are also closely related to various lung diseases such as sinusitis and chronic obstructive pulmonary disease (COPD) (Huang et al., 2017b). A study showed that sinusitis patients experienced an increase in *Corynebacterium tuberculostearicum* (Abreu et al., 2012). In COPD, increased *Lactobacillus* is induced by an inflammatory modulation and results in the formation of tertiary lymphoid (Sze et al., 2012). All the above studies revealed the close associations between microbes and various human diseases. Unquestionably, identifying potential microbe-disease associations is of great significance in exploring the pathogenesis, prevention, and treatment of diseases. As the traditional experimental method is time-consuming, costly, random, and blind, there is an urgent need to develop an effective calculation approach so as to help researchers in finding the regular pattern of microbe-disease associations and to provide complementary and supportive evidence for the experimental study.

Relevant research for the identification of potential microbe-disease associations are still in its infancy, and effective calculation models for the association prediction are even more scarce. Ma et al. (2017) created the first database of Human Microbe-Disease Association Database (HMDAD), which collected confirmed microbe-disease associations from published literatures. Based on the above work, several computational models were established to prioritize candidate microbes for diseases. For example, Chen et al. (2017a) introduced the network-based model of KATZ measure for Human Microbe-Disease Association prediction (KATZHMDA), the first calculation method for the identification of new microbe-disease associations through computing the number of walks of connections between microbe and disease nodes in the microbe-disease association network. Recently, the computational model of Laplacian Regularized Least Squares for Human Microbe-Disease Association (LRLSHMDA) was presented by Wang et al. (2017). It is a global measure based on a semi-supervised learning framework. In their proposed calculation model, the Laplacian regularized least squares (LapRLS) classification was adopted to prioritize candidate microbes for all interested diseases through the application of known microbe-disease associations, the Gaussian interaction profile kernel similarity for microbes and diseases. Similarly, with the same dataset of known microbe-disease associations, the Gaussian interaction profile kernel similarity for microbes and diseases mentioned above, a path-based search model of Path-Based Human Microbe-Disease Association prediction (PBHMDA) was introduced by Huang et al. (2017c). In the model, the association score of each microbe-disease pair would be computed by the integration of all paths less four between the microbe and disease with different weights. In addition, Huang et al. (2017a) put forward a Neighbor- and Graph-based combined Recommendation model for Human Microbe-Disease Association prediction (NGRHMDA). The final prediction scores of novel microbe-disease associations were attained via the integration of two prediction results predicted by neighbor-based collaborative filtering and the graph-based scoring method. Also, Peng et al. (2018) put forward a model of Adaptive Boosting for Human Microbe-Disease Association prediction (ABHMDA) by enforcing a strong classifier on the samples. Specifically, the strong classifier was constructed by the integration of 30 weak classifiers with different weights.

In this paper, by combining known microbe-disease associations collected from HMDAD, disease symptom similarity and Gaussian interaction profile kernel similarity for microbes and diseases, we introduced a computational model of Matrix Decomposition and Label Propagation for the Human Microbe-Disease Association prediction (MDLPHMDA). In our proposed algorithm, a new adjacency matrix of microbe-disease associations was first generated by employing the spare learning method (SLM) on the original association information extracted from HMDAD, and potential microbe-disease associations would be further predicted under the implementation of the label propagation algorithm (LPA). The leave-one-out cross validation (LOOCV) and five-fold cross validation were subsequently enforced for accuracy evaluation of MDLPHMDA. Assessment results showed that MDLPHMDA gained the area under the receiver operating characteristic curves (AUCs) of 0.9034 and 0.8954 ± 0.0030 in LOOCV and five-fold cross validation, respectively. In case studies, we carried out MDLPHMDA to predict potential microbes for asthma and colorectal carcinoma (CRC), respectively. Moreover, via the implementation of our developed algorithm, we prioritized microbes for liver cirrhosis and type 1 diabetes by removing their known related microbes, respectively. Finally, the results analysis of cross validations and case studies showed that MDLPHMDA is a suitable and effective model in potential microbe-disease association prediction.

## Materials and Methods

### Human Microbe-Disease Associations

The dataset of confirmed microbe-disease associations used in this paper were collected from HMDAD (http://www.cuilab.cn/hmdad) (Ma et al., 2017). According to the 16s RNA sequencing-based microbiome research, the database collected 483 microbe-disease associations between 39 diseases and 292 microbes from 61 previous works. Along with the deletion of the same microbe-disease associations based on different evidences in the database, we finally obtained a dataset of 450 associations between 39 diseases and 292 microbes. Moreover, the variables *nd* and *nm* were defined to represent the 39 diseases and 292 microbes, respectively. Also, adjacency matrix *A* of the verified microbe-disease associations was defined as follows:

(1) $$\begin{array}{l} A\left(i,j\right)=\{\begin{array}{c} 1,if\;microbe\;m\left(j\right)\;is\;related\;to\;disease\;d\left(i\right)\\ 0,\;otherwise \end{array} \end{array}$$

### Integrated Diseases Similarity

The integrated disease similarity was constructed by combining the Gaussian interaction profile kernel similarity for diseases and disease symptom similarity. First, we calculated the Gaussian interaction profile kernel similarity for diseases by adopting the calculation approach in the previous literature (Van Laarhoven et al., 2011). According to the idea that similar diseases possess similar interaction and non-interaction patterns with microbes, the Gaussian interaction profile kernel similarity for diseases was created in light of confirmed microbe-disease associations. We defined the interaction profile of each disease by using a binary vector that shows whether the disease is related to each microbe or not. For example, for disease *d*(*i*), its interaction profile *IP*(*d*(*i*)) is the *i*th row of the adjacency matrix *A*. Therefore, the Gaussian interaction profile kernel similarity between disease *d*(*i*) and disease *d*(*j*) can be computed as follows:

(2) $$\begin{array}{l} KD\left(d\left(i\right),d\left(j\right)\right)=\exp\left(-\gamma_{d}{\left\|IP\left(d\left(i\right)\right)-IP\left(d\left(j\right)\right)\right\|}^{2}\right) \end{array}$$

(3) $$\begin{array}{l} \gamma_{d}={\gamma_{d}}^{\prime }/\left(\frac{1}{nd}\overset{nd}{\underset{k=1}{\sum }}{\left\|IP\left(d\left(k\right)\right)\right\|}^{2}\right) \end{array}$$

where γ_*d*_ indicates the normalized kernel bandwidth in light of the new bandwidth parameter ${\gamma_{d}}^{\prime }$. Second, according to the data of diseases and their symptoms in PubMed bibliography, disease symptom similarity *DSS* could be constructed (Zhou et al., 2014). Finally, in accordance with disease symptom similarity put forward by Zhou et al. (2014), taking into account of the Gaussian interaction profile kernel similarity for diseases, we constructed integrated disease similarity by using the method applied in a previous study (Chen et al., 2017a).

(4) $$\begin{array}{l} DS=\frac{KD+DSS}{2} \end{array}$$

### Gaussian Interaction Profile Kernel Similarity for Microbes

In the same way, motivated by previous literature (Van Laarhoven et al., 2011), the Gaussian interaction profile kernel similarity for microbes was established according to confirmed microbe-disease associations. For microbe *m*(*j*), its interaction profile *IP*(*m*(*j*)) is the *j*th column of the adjacency matrix *A*. Therefore, the Gaussian interaction profile kernel similarity between microbe *m*(*i*) and microbe *m*(*j*) can be computed as follows:

(5) $$\begin{array}{l} KM\left(m\left(i\right),m\left(j\right)\right)=\exp\left(-\gamma_{m}{\left\|IP\left(m\left(i\right)\right)-IP\left(m\left(j\right)\right)\right\|}^{2}\right) \end{array}$$

(6) $$\begin{array}{l} \gamma_{m}={\gamma_{m}}^{\prime }/\left(\frac{1}{nm}\overset{nm}{\underset{k=1}{\sum }}{\left\|IP\left(m\left(k\right)\right)\right\|}^{2}\right) \end{array}$$

where γ_*m*_ indicates the normalized kernel bandwidth in light of the new bandwidth parameter ${\gamma_{m}}^{\prime }$.

### MDLPHMDA

**Figure 1:**
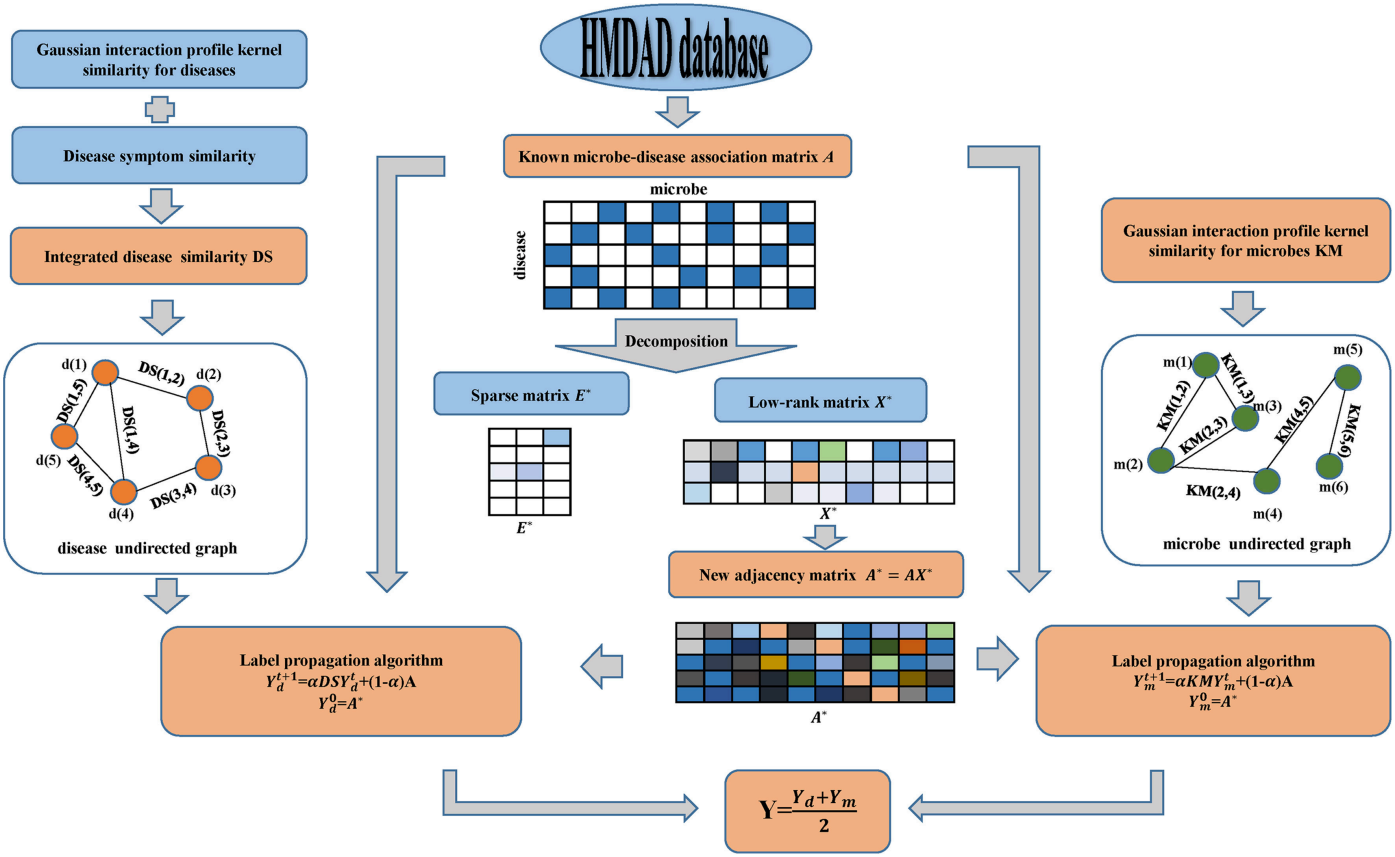
Flowchart of the calculation model of MDLPHMDA: We first enforced matrix decomposition to eliminate the noise of original known microbe-disease associations and gained a new adjacency matrix. Then LPA was implemented based on the created new adjacency matrix for the identification of the potential microbe-disease associations.

In this manuscript, motivated by SLM developed by Pech et al. (2017) and LPA introduced by Zhang et al. (2017), we applied the calculation model of MDLPHMDA to infer novel microbe-disease associations. Starting from the fact that redundant formation may be present in the original dataset of known microbe-disease associations, we employed matrix decomposition to eliminate the noise of known microbe-disease associations and then applied LPA for the identification of the potential microbe-disease associations (see Figure 1). It is worth mentioning that matrix decomposition has been widely used in Bioinformatics research (Chen et al., 2018b,d; Zhao et al., 2018).

Since a part of microbe-disease associations in the dataset may be incorrect or redundant, we adopted SLM to remove the noise of the original data and search a lowest-rank matrix among candidates to gain a novel adjacency matrix. In our introduced model, we divided the original adjacency matrix *A* into two parts by using SLM. The first part is a linear combination of the original adjacency matrix *A* and a low-rank matrix, while the second part is a spare matrix that can be regarded as the noise of the original adjacency matrix *A*. Hence, the original adjacency matrix can be decomposed as follows:

(7) $$\begin{array}{l} A=AX+E \end{array}$$

In order to get a low-rank matrix *X* and a sparse matrix *E*, we could transform Equation (7) into a optimization problem by applying the nuclear norm on *X* and the sparse norm on *E*.

(8) $$\begin{array}{l} \underset{X,E}{min}{\;\|X\|}_{*}+\alpha {\left\|E\right\|}_{2,1}\;s.t.\;A=AX+E \end{array}$$

where

(9) $$\begin{array}{l} {\left\|A\right\|}_{*}=\sum_{i}\sigma_{i}\;\left(\text{i}.\text{e}.,\sigma_{i}\text{ is the sigular values of A}\right) \end{array}$$

(10) $$\begin{array}{l} {\left\|E\right\|}_{2,1}=\sum_{j=1}^{n}\sqrt{\sum_{i=1}^{n}{\left(E_{ij}\right)}^{2}} \end{array}$$

Here, α is a positive free parameter, which can balance the weight between the low-rank matrix and the sparse matrix. To transform the original optimization problem into an augmented Lagrange function, we rewrote the optimization problem into a constraint and convex optimization problem of Equation (11) and enforced an inexact augmented Lagrange multipliers (IALM) algorithm (Meng et al., 2014) to solve it (see Table 1).

(11) $$\begin{array}{l} \underset{X,E,J}{min}{\;\|J\|}_{*}+\alpha {\left\|E\right\|}_{2,1}\\ s.t.\;A=AX\text{+}E,X=J \end{array}$$

(12) $$\begin{array}{l} L=\|J{\|}_{*}+\alpha \|E{\|}_{2,1}+tr\left(Y_{1}^{T}\left(A-AX-E\right)\right)\\ +tr\left(Y_{2}^{T}\left(X-J\right)\right)+\frac{\mu }{2}\left(\|A-AX-E{\|}_{F}^{2}+\|X-J{\|}_{F}^{2}\right) \end{array}$$

where μ ≥ 0 is a penalty parameter and the detailed solution process to gain solution X^*^ and E^*^ of Equation (12) could be explained in previous literature (Pech et al., 2017).

**Table 1:** Computational procedures of the Inexact augmented Lagrange multipliers (IALM) algorithm.

**Algorithm: IALM**
**Input:** Given a adjacency matrix **A** and parameter α = 0.1
Output:X^*^andE^*^
**Initialize:**$\text{X}=\text{0},\text{E}=\text{0},{\text{Y}}_{1}=\text{0},{\text{Y}}_{2}=\text{0,}\mu =\text{1}{\text{0}}^{-\text{4}},\max_{\mu }=\text{1}{\text{0}}^{\text{10}},\rho \;=\;\text{1}\text{.1},\varepsilon =\text{1}{\text{0}}^{-6}$
**while** ||**A** − **AX** − **E**||_∞_ ≥ ε and ||**X** − **J**||_∞_ ≥ ε **do**
a.$J=\arg\min\frac{1}{\mu }\|J{\|}_{*}+\frac{1}{2}{\left\|J-(X+Y_{2}/\mu )\right\|}_{\text{F}}^{2}$
b.$\text{X}=\left(\text{I}+{\text{A}}^{T}\text{A}\right)\left({\text{A}}^{T}\text{A}-{\text{A}}^{T}\text{E}+\text{J}+\left({\text{A}}^{T}Y_{1}-{\text{Y}}_{2}\right)/\mu \right)$
c. $E=\arg\min\frac{\alpha }{\mu }\|E{\|}_{2,1}+\frac{1}{2}{\left\|E-(A-AX+Y_{1}/\mu )\right\|}_{\text{F}}^{2}$
d.**Y**_1_ = **Y**_1_ + μ(**A** − **AX** − **E**); **Y**_2_ = **Y**_2_ + μ(**X** − **J**)
e. μ = min(ρμ, max_μ_)
end while

As the solution of Equation (12) was solved, we gained a new adjacency matrix A^*^ with less noise by the linear combination of the original adjacency matrix *A* and the low-rank matrix X^*^ as follows:

(13) $$\begin{array}{l} A^{*}=AX^{*} \end{array}$$

Then, based on the Gaussian interaction profile kernel similarity for microbes and diseases, disease symptom similarity and the newly created adjacency matrix A^*^, we enforced LPA to infer novel microbe-disease associations. First, from the perspective of disease, we constructed an undirected graph with diseases as nodes, and similarity scores as edge weight. To combine the original microbe-disease associations information, we treated the new adjacency matrix of microbe-disease associations as the labels to propagate in the disease undirected graph and each label is updated through the absorption of its neighborhoods' label information with a rate of α and going back to its original known microbe-disease association nodes with a rate of 1 − α. Referring to previous literature (Yao et al., 2017; Zhang et al., 2017), we set α as 0.3. The label propagation process can be described as follows:

(14) $$\begin{array}{l} {Y_{d}}^{t+1}=\alpha DS{Y_{d}}^{t}+\left(1-\alpha \right)A \end{array}$$

where ${Y_{d}}^{t}$ indicates the predicted scores between microbes and diseases at step *t*. Specifically, $Y_{d}^{0}$ refers to the newly created adjacency matrixA^*^. The iteration would be stable after some steps (the change in value between ${Y_{d}}^{t+1}$ and ${Y_{d}}^{t}$ measured by *L*_1_ norm is <10e-6). The final value *Y*_*d*_ would be the predicted scores of new microbe-disease associations from the perspective of diseases.

Also, from the perspective of microbes, we can build another microbe undirected graph and employ LPA to gain another predicted scores *Y*_*m*_ of novel microbe-disease associations. Finally, we defined the final predicted scores *Y* for the potential microbe-disease associations by the average of the two predicted scores mentioned above.

(15) $$\begin{array}{l} Y=\frac{Y_{d}+Y_{m}}{2} \end{array}$$

## Results

### Performance Evaluation

**Figure 2:**
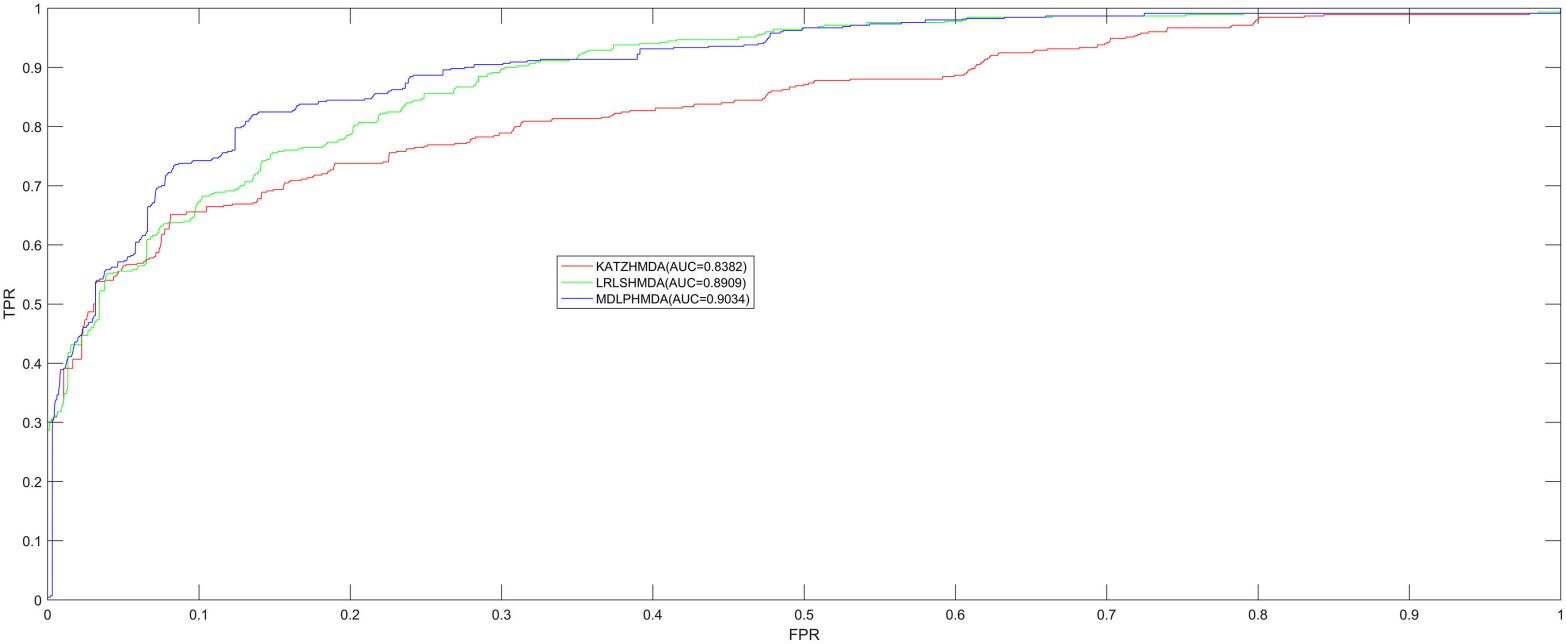
Performance comparison between MDLPHMDA and other two classical microbe-disease association prediction models (LRLSHMDA and KATZHMDA) by means of AUCs based on LOOCV. The results showed that MDLPHMDA gained AUCs of 0.9034 in LOOCV.

In order to test the prediction performance of MDLPHMDA based on the 450 confirmed microbe-disease associations collected from HMDAD (Ma et al., 2017), our model was compared with two classic algorithms (LRLSHMDA and KATZHMDA) on the basis of the evaluation method of LOOCV and five-fold cross validation. In LOOCV, each confirmed microbe-disease association was taken as test sample by turn and the rest 449 identified associations were used to train. After executing MDLPHMDA, the score of the test sample would be ranked with the scores of candidate samples that were made up of all unconfirmed microbe-disease pairs. In five-fold cross validation, we first divided the 450 microbe-disease association pairs into five equal parts and later made each part as test sample in turn and the remaining four parts of associations as training samples. In the same way, each test sample's score would be ranked with the scores of all candidate samples that were composed of unconfirmed microbe-disease pairs. As the sample divisions may cause bias, we enforced five-fold cross validation 100 times to gain an average value as the final result. If the ranking of the test sample is higher than a given threshold, our model is considered to make a successful prediction. Then, according to varying thresholds, we plotted the receiver operating characteristics (ROC) curve by computing the ratio of true positive rate (TPR, sensitivity) to false positive rate (FPR, 1-specificity). Sensitivity denotes the percentage of test samples which obtained ranks higher than the set threshold. Meanwhile, specificity denotes the percentage of negative microbe-disease pairs with ranks lower than the threshold. Finally, to assess the performance of MDLPHMDA effectively, we computed corresponding AUCs. When AUC = 1, the model possesses perfect forecast ability; when AUC = 0.5, the model possesses random forecast ability. In LOOCV, assessment results showed that MDLPHMDA, LRLSHMDA, and KATZHMDA gained the AUCs of 0.9034, 0.8909, and 0.8382, respectively (see Figure 2). In five-fold cross validation, MDLPHMDA, LRLSHMDA, and KATZHMDA gained the AUCs of 0.8954 ± 0.0030, 0.8794 ± 0.0029, and 0.8301 ± 0.0033, respectively. Stated thus, it can be seen that our model possesses good prediction ability and could be used to assist the identification of novel microbe-disease associations. Moreover, we carried out a paired *t*-test based on the ranking results of LOOCV to observe the statistical significance of differences among MDLPHMDA, LRLSHMDA, and KATZHMDA. As a result, the *p*-value of MDLPHMDA and LRLSHMDA is 0.0088, whereas the *p*-value of MDLPHMDA and KATZHMDA is 1.2510e-08. We can see that MDLPMDA is significantly different from LRLSHMDA and KATZHMDA on the basis of their ranking results of LOOCV (*p* < 0.05).

### Case Study

Via two different types of case studies, we further assessed the prediction ability of MDLPHMDA based on the confirmed 450 microbe-disease associations. In the first kind, we identified potential microbes for asthma and CRC, respectively, through the implementation of MDLPHMDA. Also, we released all prediction scores for 10938 novel microbe-disease pair between 39 diseases and 292 microbes (see Supplementary Table 1). In the second kind, we enforced MDLPHMDA to identify liver cirrhosis-associated microbes by removing 62 known liver cirrhosis-associated microbes from the dataset of known microbe-disease associations and also predicted for another disease of type 1 diabetes by removing its known microbes. Based on the results of the two types of case studies, the proposed algorithm of MDLPHMDA was proven to be an effective algorithm in the identification of novel microbe-disease associations.

Asthma is a long-term inflammatory disease of the airways (Lemanske and Busse, 2010). Its common symptoms include coughing, reversible airflow obstruction, wheezing, or bronchospasm (Lemanske and Busse, 2010). Epidemiological studies indicated that microbial exposures in early life might determine microbiota composition, which can help to prevent allergy or lead to the development of asthma (Wang et al., 2003; Weber et al., 2015). A study in asthmatic children has found a low abundance of *Bifidobacterium* in their intestinal microbiota, which may reduce the immune function and potentially contribute to disease chronicization (Kalliomaki et al., 2001). Similarly, a probiotic strain *Lactobacillus rhamnosus* reduced allergic responses in the airways of neonates (Martinon et al., 2009). In this paper, via the implementation of MDLPHMDA for the inference of novel asthma-related microbes, we could see that the top 10 predicted microbes for asthma were all confirmed through literature (see Table 2). Among the top 3 confirmed associations between microbes and asthma, relevant differences in *Firmicutes* were found between samples from asthmatic and non-asthmatic subjects (Marri et al., 2013). Another study investigated that *Clostridium difficile* was associated with an increased risk for asthma (Van Nimwegen et al., 2011). Meanwhile, in a study about early intestinal colonization of infants, *Clostridium coccoides* was confirmed to be associated with increased risk for the development of asthma before the age of 3 years (Vael et al., 2011).

**Table 2:** The validation of the top 10 predicted asthma-related microbes after implementing MDLPHMDA based on the confirmed microbe-disease associations from HMDAD.

**Disease**	**Microbe**	**Score**	**Evidence**
Asthma	*Firmicutes*	0.035113958	PMID:23265859
Asthma	*Clostridium difficile*	0.026401159	PMID:21872915
Asthma	*Clostridium coccoides*	0.023247917	PMID:21477358
Asthma	*Staphylococcus aureus*	0.022891159	PMID:25533526
Asthma	*Actinobacteria*	0.022703105	PMID:23265859
Asthma	*Lachnospiraceae*	0.022512904	PMID: 27433177
Asthma	*Lactobacillus*	0.022019371	PMID:20592920
Asthma	*Enterobacteriaceae*	0.018834777	PMID:21639872
Asthma	*Veillonella*	0.018243753	PMID: 26424567
Asthma	*Bacteroides*	0.017354957	PMID: 18822123

*As a result, all of the top 10 predicted microbes were confirmed by literatures*.

CRC is the cancer in the colon or rectum (Watson and Collins, 2011). Common symptoms include weight loss, blood in stool, and feeling tired all the time (Watson and Collins, 2011). It typically starts in the form of a polyp as a benign tumor, which becomes cancerous over time (Watson and Collins, 2011). A quantitative polymerase chain reaction (qpcr) analysis verified that *Fusobacterium nucleatum*, an invasive anaerobe previously linked to appendicitis and periodontitis but not to cancer, was increased in a CRC tumor vs. normal tissue (Castellarin et al., 2012). Furthermore, this overabundance is positively associated with lymph node metastasis (Castellarin et al., 2012). Another study also observed a significant difference of *Bacteroides* and *Prevotella* in a CRC group, as compared to a normal group (Sobhani et al., 2011). Moreover, we employed the proposed algorithm to predict CRC-related microbes and the outcomes displayed that all but one of the top 10 microbes for CRC were verified (see Table 3). Among the top 3 confirmed associations, according to the taxonomic results, *Proteobacteria* showed a higher abundance in CRC rats compared to control groups and constitute the third most abundant phyla (Zhu et al., 2014). In another analysis on CRC, the *Helicobacter pylori* infection was noted in 50 CRC patients. Furthermore, an infection with *H. pylori CagA*+ was associated with an increased risk for CRC (Shmuely et al., 2001). Moreover, a statistically significant difference in *C. difficile* was detected between the CRC and healthy group, suggesting a possible role of this bacteria in CRC carcinogenesis (Fukugaiti et al., 2015).

**Table 3:** The validation of the top 10 predicted CRC-related microbes after implementing MDLPHMDA based on the confirmed microbe-disease associations from HMDAD.

**Disease name**	**Microbe name**	**Score**	**Evidence**
CRC	*Proteobacteria*	0.046859257	PMID: 24603888
CRC	*Helicobacter pylori*	0.023587271	PMID: 11774957
CRC	*Clostridium difficile*	0.023311807	PMID: 26691472
CRC	*Actinobacteria*	0.023310463	Unconfirmed
CRC	*Lactobacillus*	0.022992627	PMID:15828052
CRC	*Haemophilus*	0.022582968	PMID:26549775
CRC	*Lachnospiraceae*	0.02243978	PMID:21850056
CRC	*Clostridium coccoides*	0.021696923	PMID:18237311
CRC	*Enterobacteriaceae*	0.021122176	PMID: 25182170
CRC	*Staphylococcus aureus*	0.020450372	PMID:7074582

*As a result, 9 out of the top 10 predicted microbes were confirmed by literatures*.

Liver cirrhosis is a disease induced by long-term damage. This damage is due to the replacement of normal tissue by scar tissue (Li et al., 1999). Typically, the disease develops slowly and there are often no significant early symptoms. As it worsens, patients may become tired, bruise easily, develop yellow skin, have fluid in the abdomen, or have swelling in the lower legs (Li et al., 1999). Liver cirrhosis is commonly caused by alcohol, non-alcoholic fatty liver disease, hepatitis B, or hepatitis C (Li et al., 1999). In a study on the alterations of the human microbiome in liver cirrhosis, quantitative metagenomics reveals 66 cognate bacterial species that differ in abundance between healthy individuals and patients, including *Alistipes finegoldii, Bacteroides eggerthii, Eubacterium rectale, Faecalibacterium prausnitzii, Haemophilus parainfluenzae*, and so on (Qin et al., 2014). In another study about fecal microbial communities in patients with liver cirrhosis, research has detected the prevalence of pathogenic bacteria such as *Enterobacteriaceae* and *Streptococcaceae* as well as the reduction of beneficial populations such as *Lachnospiraceae* (Chen et al., 2011). Here, by removing 62 known liver cirrhosis-associated microbes from the dataset of known microbe-disease associations, we enforced MDLPHMDA to identify liver cirrhosis-associated microbes on the basis of integrated disease similarity, Gaussian interaction profile kernel similarity for microbes, and the rest known microbe-disease associations. As a result, 9 out of the top 10 microbes for liver cirrhosis were confirmed by HMDAD and literature (see Table 4). Among the top 3 confirmed associations, *Firmicutes* was found to be highly enriched in the patients group (Chen et al., 2011). Moreover, researchers found significantly higher *H. pylori* prevalence in patients with previous hospital admissions (Siringo et al., 1997). This high prevalence of *H. pylori* is related to age and sex (Siringo et al., 1997). An analysis on the *C. difficile* infection in patients with liver cirrhosis showed that cirrhotic patients with the *C. difficile* infection have increased mortality than those without the *C. difficile* infection, suggesting the importance of *C. difficile* in the diagnosis and therapy of liver cirrhosis (Trifan et al., 2015).

**Table 4:** The validation of the top 10 predicted liver cirrhosis-associated microbes after implementing MDLPHMDA by removing liver cirrhosis-related associations from the dataset of known microbe-disease associations.

**Disease name**	**Microbe name**	**Score**	**Evidence**
Liver cirrhosis	*Proteobacteria*	0.037652208	HMDAD
Liver cirrhosis	*Bacteroidetes*	0.033708121	HMDAD
Liver cirrhosis	*Firmicutes*	0.033302216	PMID:21574172
Liver cirrhosis	*Prevotella*	0.028842704	HMDAD
Liver cirrhosis	*Helicobacter pylori*	0.021108828	PMID:9365129
Liver cirrhosis	*Clostridium difficile*	0.020872569	PMID:26440041
Liver cirrhosis	*Actinobacteria*	0.020204542	PMID:22326468
Liver cirrhosis	*Clostridium coccoides*	0.018391455	Unconfirmed
Liver cirrhosis	*Staphylococcus aureus*	0.01835198	PMID:22833245
Liver cirrhosis	*Lactobacillus*	0.016449739	HMDAD

*As a result, 9 out of the top 10 predicted microbes were confirmed by HMDAD and literatures*.

Type 1 diabetes is a type of diabetes mellitus induced by very little or no insulin produced in the pancreas (Daneman, 2006). It results in high blood sugar levels in the human body. The classic symptoms include increased thirst and hunger, frequent urination and weight loss (Daneman, 2006). The cause of type 1 diabetes is still unclear. However, it is believed to involve both genetic and environmental factors (Chiang et al., 2014). One theory proposes that type 1 diabetes may be caused by an autoimmune response while the immune system attacks virus-infected insulin-producing cells in the pancreas (Knip et al., 2005). In a microbiome metagenomics analysis on type 1 diabetes, researchers identified the differences between patients and controls at the genus level. The most significant differences were noted in the genera *Prevotella* and *Bacteroides* (Brown et al., 2011). In another study defining the autoimmune microbiome for type 1 diabetes, scientists identified bacteria that correlated with the autoimmune state including *Bacteroides fragilis, Clostridia, Eubacterium eligens*, and so on (Giongo et al., 2011). Similarly, we employed MDLPHMDA to identify type 1 diabetes-associated microbes by removing 167 known type 1 diabetes-associated microbes from the dataset of known microbe-disease associations. The results showed that 8 out of the top 10 microbes for liver cirrhosis were confirmed (see Table 5). In a case-control study, scientists found a meaningful correlation between the *H. pylori* infection and the duration of diabetes in type 1 diabetic children (Bazmamoun et al., 2016). In another study, researchers found that *Staphylococcus aureus* is associated with the vitamin D receptor (VDR) polymorphisms in patients with type 1 diabetes (Panierakis et al., 2009).

**Table 5:** The validation of the top 10 predicted type 1 diabetes-related microbes after implementing MDLPHMDA by removing type 1 diabetes-related associations from the dataset of known microbe-disease associations.

**Disease name**	**Microbe name**	**Score**	**Evidence**
Type 1 diabetes	*Proteobacteria*	0.035314225	HMDAD
Type 1 diabetes	*Bacteroidetes*	0.030725459	HMDAD
Type 1 diabetes	*Firmicutes*	0.02813792	HMDAD
Type 1 diabetes	*Prevotella*	0.026244828	HMDAD
Type 1 diabetes	*Clostridium difficile*	0.021633674	Unconfirmed
Type 1 diabetes	*Helicobacter pylori*	0.021284419	PMID:27497772
Type 1 diabetes	*Clostridium coccoides*	0.019300705	Unconfirmed
Type 1 diabetes	*Staphylococcus aureus*	0.017744364	PMID:19411183
Type 1 diabetes	*Lactobacillus*	0.016082255	HMDAD
Type 1 diabetes	*Actinobacteria*	0.015923043	HMDAD

*As a result, 8 out of the top 10 predicted microbes were confirmed by HMDAD and literatures*.

## Discussion

Since the application of traditional experimental methods to prioritize disease-associated microbes is time consuming and expensive, the calculation approach of MDLPHMDA was put forward through the fusing of integrated disease similarity, Gaussian interaction profile kernel similarity for microbes and known microbe-disease associations. The performance of MDLPHMDA was tested using cross validations and case studies. Results on the basis of confirmed microbe-disease associations showed that the performance of our introduced algorithm is significantly improved in contrast with other two classic algorithms of LRLSHMDA and KATZHMDA. Consequently, the introduced algorithm is a suitable and effective model in the identification of novel microbe-disease associations. We further expect that the identified microbe-disease associations with high probability scores would be verified through biological experiment in the future.

The reason why MDLPHMDA could get excellent prediction performance is due to the following attractive properties. First, with the application of SLM on the original information of known microbe-disease associations, a new adjacency matrix with more accurate association information (the linear combination of low-rank matrix and the original adjacency matrix) and a noise (sparse) matrix would be gained. Obviously, in light of the new generated adjacency matrix, the forecast performance of the proposed algorithm for the identification of new microbe-disease associations could be significantly enhanced. Second, LPA was used to predict novel microbe-disease associations from the perspectives of microbe and disease, respectively, which would promote the ability of MDLPHMDA in terms of forecast accuracy. Third, in comparison with the previous calculation algorithms that only used Gaussian interaction profile kernel similarity for diseases as disease similarity, MDLPHMDA could achieve superior performance through integrating disease symptom similarity and Gaussian interaction profile kernel similarity for diseases into the final disease similarity. Moreover, the implementation of MDLPHMDA does not require negative samples and the algorithm could be applied to new diseases (microbes) without the relevant microbes (diseases).

However, the model has some main disadvantages. For instance, the amount of known microbe-disease associations used in this paper is very finite and more confirmed microbe-disease associations need to be collected. Additionally, as the computation of Gaussian interaction profile kernel similarity of microbes depended on known microbe-disease associations, other features of microbe similarity should be collected and combined to gain a more comprehensive dataset of microbe similarity such as microbe-drug associations collected by MDAD (Sun et al., 2018). For MDLPHMDA, it is difficult to find the optimum value of all the parameters to ensure that the prediction model achieves the highest accuracy. Also, the employment of SLM for creating new adjacency matrix may bring unnecessary and useless association information, which would affect the prediction result of LPA. Finally, successfully established models in the other computational fields would inspire the development of microbe-disease association prediction, such as microRNA-disease association prediction (Chen and Huang, 2017; Chen et al., 2018c), long non-coding RNA-disease association prediction (Chen and Yan, 2013; Chen et al., 2017b), drug-target interaction prediction (Chen et al., 2016b, 2018a), and synergistic drug combinations (Chen et al., 2016a).

## Author Contributions

JQ developed the prediction method, implemented the experiments, analyzed the result, and wrote the paper. YZ conceived the project, designed the experiments, analyzed the result, and revised the paper. JY analyzed the result and revised the paper.

### Conflict of Interest Statement

The authors declare that the research was conducted in the absence of any commercial or financial relationships that could be construed as a potential conflict of interest.
